# Atypical Cystic Fibrosis: Diagnosis at the Age of 57 Years

**DOI:** 10.7759/cureus.10863

**Published:** 2020-10-09

**Authors:** Gabrielle J Sagesse, Sanjay Yadava, Anupa Mandava

**Affiliations:** 1 Internal Medicine, State University of New York Upstate Medical University and the Syracuse Veterans Affairs Medical Center, Syracuse, USA

**Keywords:** cystic fibrosis, atypical cystic fibrosis, cf, pulmonary, pneumonia, bronchiectasis, cftr, pseudomonas aeuginosa, fat malabsorption

## Abstract

Cystic fibrosis (CF) is an autosomal recessive, multi-organ disorder found predominantly among Caucasians. It classically presents in childhood with chronic productive cough, malabsorption causing steatorrhea, and failure to thrive. We present a 75-year-old female diagnosed with CF at the age of 57 years, which highlights the natural history and challenges in the diagnosis of atypical CF, including broadening physicians’ respiratory differential diagnosis, limited patient symptoms, and late age of symptom onset.

## Introduction

Cystic fibrosis (CF) is a complex, autosomal recessive exocrinopathy affecting multiple organs. It classically presents within the first few years of life with pulmonary disease, pancreatic insufficiency, malabsorption, malnutrition, and diagnosed with a positive sweat chloride screening test. There exists another clinically diverse group of individuals with cystic fibrosis who usually have onset in adulthood with minimal symptoms involving only a single organ, or more, and a negative sweat chloride test called atypical CF. The diagnosis of atypical CF poses a challenge for clinicians due to its unusual presentation and late-onset of symptoms. The life expectancy of individuals with atypical CF is typically longer than for individuals with classic CF; however, the long-term outcomes of atypical CF remain unknown. In this case report, we present a 75-year-old female diagnosed with atypical cystic fibrosis at an age of 57 years which highlights the natural history, challenges in diagnosis, and resulting treatment and outcome. 

## Case presentation

As a child, the patient reported a history of seasonal allergies, bronchitis (one episode), chronic sinusitis (one episode yearly), latent TB with a purified protein derivative (PPD) conversion at age 19 years, and multiple nasal polyps that were surgically removed (last removal 15 years ago). She smoked half a pack of cigarettes per day for 35 years and was exposed to second-hand smoke from both her parents as a child. Otherwise, past medical history was noncontributory. Her family history includes chronic obstructive pulmonary disease in both her mother and brother. Her brother also had late-onset CF, diagnosed at the age of 72 years.

At age 57 years, the patient began developing recurrent bronchitis and pneumonia which led the patient to undergo computed tomography (CT) scan of her chest and pulmonary function testing (PFT) as recommended by her pulmonologist. The patient’s CT scan revealed diffuse upper lobe-predominant bronchiectasis, however, her PFT revealed normal spirometry. She also had multiple sputum cultures which repeatedly grew Pseudomonas. Given the patient’s finding of bronchiectasis in her CT chest with a normal PFT, recurrent Pseudomonas chest infection, and positive family history of CF, she was recommended for CF genetic testing, which revealed two separate mutations: a deletion in deltaF508 and Nt 3599 +1 change from G → A. She also underwent sweat chloride testing which revealed an elevated level of 90 mEq/L. Another workup for the classical manifestation of CF in this patient including malabsorption, pancreatic insufficiency, hepatobiliary, and genitourinary was negative. The patient was subsequently diagnosed with atypical cystic fibrosis at 57 years. Other microorganisms that have affected the patient since her time of diagnosis include *Citrobacter freundii*, *Alcaligenes xylosoxidans*, *Burkholderia multivorans*, subspecies bolletii, *Mycobacterium avium* complex, and *M. abscessus*. The patient has never required IV antibiotics or been hospitalized for CF exacerbation until July 2019 when her care was transferred to Upstate Medical University. The patient recalls remaining healthy and very active previously.

**Figure 1:**
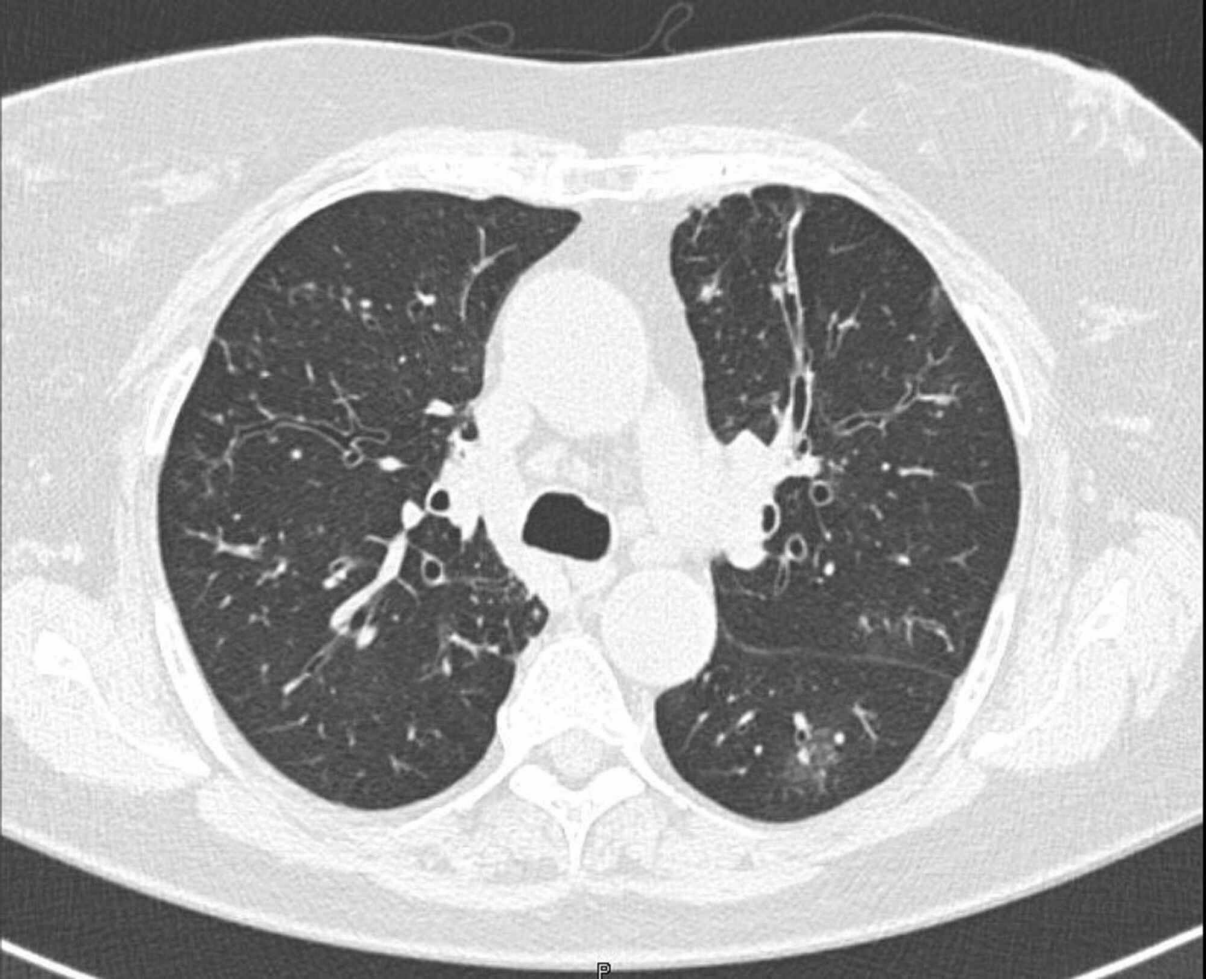
CT chest of the patient taken in August 2019 at Upstate Medical University See multifocal airspace opacities and diffuse upper lobe predominant bronchiectasis.

At age 75 years, a second CT chest was performed at Upstate in August 2019 revealing a worsening clinical picture with multifocal airspace opacities (likely representing multifocal pneumonia) and diffuse upper lobe predominant bronchiectasis, consistent with the patient's known cystic fibrosis (Figure 1).

In September 2019, the patient presented to Upstate with a two-month history of productive cough, shortness of breath, decreased energy, and persistent cultures positive for nontuberculous *M. abscessus* (NTM) infections. On physical examination, the patient was generally well-appearing. Head, eyes, ears, nose, and throat (HEENT) examination revealed no nasal polyps, nasal turbinate hypertrophy, or tenderness upon palpation of the sinuses. On respiratory examination, the patient had a productive cough with green sputum and shortness of breath, but no wheezing. Extremities showed no clubbing. The abdominal examination did not reveal increased girth, distension, or tenderness. She was admitted for the initiation of IV antibiotics to eradicate *M. abscessus* thought to be causing her symptoms. The patient was treated with hypertonic saline NaCl 3% inhalation solution, dornase alfa (pulmozyme) inhalation, azithromycin PO, amikacin, and tigecycline. Currently, she has been doing well. 

## Discussion

CF is the most commonly diagnosed genetic disorder affecting Caucasians [1]. However, not every case of CF presents with meconium ileus in the newborn, failure to thrive, or severe lung disease. Atypical CF is characterized by a milder form of the disease usually remaining undiagnosed for years, even into late adulthood [2]. Atypical cystic fibrosis may have single organ involvement, such as mild lung disease, nasal polyposis, recurrent pancreatitis, biliary cirrhosis, portal hypertension, or obstructive azoospermia compared to multiorgan involvement in classical CF [3]. Our case presentation demonstrated a patient with predominantly nasal polyposis and sinusitis in childhood and recurrent bronchitis in adulthood. Our patient had one to two organs affected as a result of her cystic fibrosis mutations. Other patients with atypical cystic fibrosis can also manifest with other single organ involvement like idiopathic chronic pancreatitis (ICP). ICP is one diagnosis that also represents the milder manifestations of cystic fibrosis transmembrane conductance regulator (CFTR) mutations resulting in partial CFTR function and subsequent single organ involvement [4,5]. 

Interestingly, patients diagnosed with atypical/mild cystic fibrosis do not have classical symptoms but might have developed bronchiectasis and advanced lung disease on an imaging study, albeit stable spirometry, similarly to our patient. Hence, early detection and initiation of respiratory therapy are important factors in slowing down the progression of lung dysfunction and decrease exacerbations [6,7].

Our patient also developed recurrent yearly bronchiectasis before she was diagnosed with atypical CF. This could have been slowed down, or prevented, if there was a high index of clinical suspicion when she presented with recurrent nasal polyps and sinusitis, in her earlier years. In a study by Ziedalski et al. [8], only 14% of adults diagnosed with CF had elevated sweat chloride above 60 mEq/dL (diagnostic), 16% had between 40 and 60 mEq/dL, and the rest had below 40 mEq/dL who were diagnosed with genetic testing and compatible clinical findings. Hence, sweat chloride testing does seem not to be a very sensitive test in the adult population for the diagnosis of late-onset CF. 

The DeltaF508 mutation commonly found in CF, accounts for 70% of CF chromosomes worldwide, however, more than 850 mutant alleles have been reported to the CF Genetic Analysis Consortium. In the adult population with CF prevalence, the prevalence of NTM infection, particularly with *M. abscessus* (which our patient had), ranges from 4 to 14% [9]. Thus, the relationship between NTM and CF should be considered by clinicians when considering atypical CF as a diagnosis.

## Conclusions

In conclusion, adult patients with single organ involvement, such as mild lung disease, recurrent sinus infections, or nasal polyps should be suspected of having atypical CF along with a differential of asthma and other chronic respiratory illnesses. It is important to include genetic testing for CF as part of the initial workup when there is clinical suspicion, especially in the adult population who tend to present with milder symptoms and fewer hospitalizations than individuals with typical CF. Furthermore, educating patients about their atypical CF symptomatology and lifestyle modifications, including possible family planning counseling, will be important aspects of this diagnosis. The long-term complications of atypical CF currently remain largely unknown, however, those with atypical CF are known to have longer life expectancies than those with typical CF. Thus, based on what we know about typical CF complications, optimizing the health of patients diagnosed with atypical CF should be prioritized by healthcare providers.

## References

[1] Gan KH, Geus WP, Bakker W, Lamers CB, Heijerman HG (1995). Genetic and clinical features of patients with cystic fibrosis diagnosed after the age of 16 years. Thorax.

[2] Schram CA (2012). Atypical cystic fibrosis: identification in the primary care setting. Can Fam Physician.

[3] Kerem E (2006). Atypical CF and CF related diseases. Paediatr Respir Rev.

[4] Chang MC, Chang YT, Wei S (2007). Spectrum of mutations and variants/haplotypes of CFTR and genotype-phenotype correlation in idiopathic chronic pancreatitis and controls in Chinese by complete analysis. Clin Genet.

[5] Noone PG, Knowles MR (2001). “CFTR-opathies”: disease phenotypes associated with cystic fibrosis transmembrane regulator gene mutations. Respir Res.

[6] Judge EP, Dodd JD, Masterson JB, Gallagher CG (2006). Pulmonary abnormalities on high-resolution CT demonstrate more rapid decline than FEV1 in adults with cystic fibrosis. Chest.

[7] Quan JM, Tiddens HA, Sy JP (2001). A two-year randomized, placebo-controlled trial of dornase alfa in young patients with cystic fibrosis with mild lung function abnormalities. J Pediatr.

[8] Ziedalski TM, Kao PN, Henig NR, Jacobs SS, Ruoss SJ (2006). Prospective analysis of cystic fibrosis transmembrane regulator mutations in adults with bronchiectasis or pulmonary nontuberculous mycobacterial infection. Chest.

[9] Esther CR Jr, Esserman DA, Gilligan P, Kerr A, Noone PG (2010). Chronic Mycobacterium abscessus infection and lung function decline in cystic fibrosis. J Cyst Fibros.

